# Primary mesenchymal stromal cells in co-culture with leukaemic HL-60 cells are sensitised to cytarabine-induced genotoxicity, while leukaemic cells are protected

**DOI:** 10.1093/mutage/geab033

**Published:** 2021-09-10

**Authors:** Liana E Gynn, Elizabeth Anderson, Gareth Robinson, Sarah A Wexler, Gillian Upstill-Goddard, Christine Cox, Jennifer E May

**Affiliations:** 1 Centre for Research in Biosciences, Department of Applied Sciences, University of the West of England, Coldharbour Lane, Bristol BS16 1QY, UK; 2 Department of Oncology/Haematology, Royal United Hospitals Bath NHS Foundation Trust, Bath BA1 3NG, UK

## Abstract

Tumour microenvironments are hallmarked in many cancer types. In haematological malignancies, bone marrow (BM) mesenchymal stromal cells (MSC) protect malignant cells from drug-induced cytotoxicity. However, less is known about malignant impact on supportive stroma. Notably, it is unknown whether these interactions alter long-term genotoxic damage in either direction. The nucleoside analogue cytarabine (ara-C), common in haematological therapies, remains the most effective agent for acute myeloid leukaemia, yet one-third of patients develop resistance. This study aimed to evaluate the bidirectional effect of MSC and malignant cell co-culture on ara-C genotoxicity modulation. Primary MSC, isolated from patient BM aspirates for haematological investigations, and malignant haematopoietic cells (leukaemic HL-60) were co-cultured using trans-well inserts, prior to treatment with physiological dose ara-C. Co-culture genotoxic effects were assessed by micronucleus and alkaline comet assays. Patient BM cells from chemotherapy-treated patients had reduced *ex vivo* survival (*P* = 0.0049) and increased genotoxicity (*P* = 0.3172) than untreated patients. It was shown for the first time that HL-60 were protected by MSC from ara-C-induced genotoxicity, with reduced MN incidence in co-culture as compared to mono-culture (*P* = 0.0068). Comet tail intensity also significantly increased in ara-C-treated MSC with HL-60 influence (*P* = 0.0308). MSC sensitisation to ara-C genotoxicity was also demonstrated following co-culture with HL60 (*P* = 0.0116), which showed significantly greater sensitisation when MSC-HL-60 co-cultures were exposed to ara-C (*P* = 0.0409). This study shows for the first time that malignant HSC and MSC bidirectionally modulate genotoxicity, providing grounding for future research identifying mechanisms of altered genotoxicity in leukaemic microenvironments. MSC retain long-term genotoxic and functional damage following chemotherapy exposure. Understanding the interactions perpetuating such damage may inform modifications to reduce therapy-related complications, such as secondary malignancies and BM failure.

## Introduction

Disease progression mediated by the bone marrow (BM) microenvironment is a recognised contributor in many haematological malignancies (HMs) (1–3). Mesenchymal stromal cells (MSC) play a critical role in haematopoiesis within this microenvironment, by providing both a physical supportive infrastructure and by secretion of cytokines, growth factors and adhesion proteins for haematopoietic stem cells (HSC) (2,4). It is therefore also permissive of the progression and protection of HMs—which develop through transformation of HSC—in its ideal, nutrient-rich environment (5–8).

HMs encompass many complex disorders, for which biological and clinical heterogeneity present great difficulties in diagnosis and treatment, compounding diverse clinical outcomes (9,10). A shared feature of all HMs is their proximity within the BM microenvironment and therefore the interaction with MSC (1,5). Commonly, these malignancies are characterised by accumulation of dysfunctional, immature leukocytes/HSC in the BM and peripheral blood, hindering the proliferation of normal mature haematopoietic cells (11). Studies have revealed abnormal BM niche properties in many of these cancers (12), including leukaemia (13–15), multiple myeloma (MM) (16,17) and lymphoma (18,19). However, this crosstalk is still poorly understood and requires further investigation, especially since extremely poor clinical outcomes are attributed to intolerance to treatment toxicity in less biologically fit patients, chemoresistance (20,21) and perhaps the tumour microenvironment.

Up to one-half of patients with acute myeloid leukaemia (AML), for example, do not show continued response to the mainstay of leukaemia therapies, cytarabine (ara-C) (22,23). Ara-C is a widely utilised clastogenic agent that induces chromosomal aberrations and inhibits normal DNA repair mechanisms (24). Genotoxicity by previous treatment such as ara-C, or interactions with an aberrant BM microenvironment, may be implicated in post-treatment BM failure after allogeneic stem cell transplantation (alloSCT) (25), and the higher incidences of secondary malignancies seen in patients treated years previously (26).

Secondary malignancies are a common complication of HM therapies (27–30), including alkylating agents, anthracyclines, topoisomerase inhibitors (31), antimetabolite drugs and radiotherapy (27). The incidence of secondary solid tumours in those treated with high-dose ara-C was greater than 2-fold at 10 years, as compared to those treated without high-dose ara-C (32). Additionally, AML/myelodysplastic syndrome (MDS) were more common in HM patients who received autologous SCT than those treated without SCT (28), explained by preparative myeloablation by total body irradiation (28). Additionally, the risk of second malignancies was shown to increase with rituximab-based treatment stage in follicular lymphoma patients (29). This may be attributed to the lasting genotoxic damage that chemo/radiotherapy produces; with MSC shown to retain long-term genotoxic and functional damage years following chemotherapy (33,34). Importantly, MSC remain of host-origin post-alloSCT, rather than replacement by donor cells following myeloablation (35). MSC are, therefore, susceptible to DNA damage accumulation/genomic instability due to prolonged lifespan, and mutations from these previous exposures (36); enabling characteristics for further tumourigenesis (37).

Numerous studies have shown that leukaemic cells co-cultured with stromal cells are less sensitive to the cytotoxic effects of chemotherapy, with increased apoptosis avoidance (38,39), increased leukaemic cell growth (40,41) and altered phenotypic expression (2,13,42). Other studies have also shown that MSC in patients with a HM are functionally altered (13,43,44); however, there is significantly less research focus on the impact of disease on the BM stroma itself.

Despite the potential gravity of lasting genotoxic damage in the clinical setting, there are no known studies to date which focus on the bidirectional modification of genotoxicity in response to treatment by the leukaemic microenvironment. The complex nature of haematopoietic disease, the BM microenvironment itself and their understudied relationship warrants further research. Better understanding of these mechanisms could improve the long-term recovery and complications caused by malignancy and chemotherapeutic treatments to the BM. This pilot study, therefore, aimed to evaluate the effect of co-culture of heterogeneous primary BM–MSC (from patients recently diagnosed either with a primary HM or subsequent to prior chemotherapy) and malignant HSC on genotoxicity, in both cell types post-ara-C exposure.

## Materials and Methods

### Cell culture

#### Sample collection.

BM aspirate samples were obtained from patients undergoing routine investigations for the diagnosis or monitoring of a haematological disease at the Royal United Hospitals NHS Foundation Trust, Bath, following donor consent and NHS ethics approval (18/NI/0036). BM aspiration was performed by clinical professionals from the iliac crest and collected into sterile lithium heparin sample tubes. Details of samples for co-culture analyses in this study are shown in Table 1, with full sample details shown in Supplementary Table I, available at *Mutagenesis* Online.

**Table 1.** Summarised characteristics of patients from which BM samples were used for co-cultures

Characteristic	*n* = 22	%
Age (years)		
Median (range)	68.5 (36–90)	
Gender		
Female:male	11:11	50:50
Treatment		
Untreated:treated	18:4	82:18
Diagnosis		
AML (at diagnosis)	3	13.6
AML (post-treatment)	2	9.1
Other HM (at diagnosis)	13	59.1
Other HM (post-treatment)	2	9.1
No HM	2	9.1

HM, haematological malignancy.

#### Mononuclear cell isolation.

The mononuclear cell (MNC) fraction was isolated from BM aspirates by density gradient centrifugation, as previously described (34). Samples were diluted 1:1 with low glucose (LG; 1,000 mg/l) Dulbecco’s Modified Eagle’s Medium (DMEM) before gentle layering onto an equal volume of Histopaque-1077 (density 1.077 g/ml). Cells were centrifuged at 600 × *g* for 20 min without brakes. MNC fraction was washed in MSC medium (LG-DMEM supplemented with 10% foetal bovine serum (FBS; STEMCELL Technologies, Cambridge, UK) and 2 mM l-glutamine). Residual erythrocytes were removed by resuspension in red cell lysis buffer (150 mM NH_4_Cl, 100 mM NaHCO_3_, 1.3 mM EDTA) for 5 min and washed in MSC medium.

#### MSC culture.

MNC were seeded at 4 × 10^5^/cm^2^ in MSC medium and incubated at 37°C/5% CO_2_. After 3 days, non-adherent haematopoietic cells were replaced with fresh medium, with cultures maintained by weekly demi-depletion until 70% confluency was reached. Cells were passaged with trypsin (0.05% trypsin/0.02% EDTA) and reseeded at 4 × 10^3^/cm^2^ (passage one; P1) and for all further passages. Cells were cryopreserved (MSC medium containing 25% FBS and 10% DMSO) in liquid nitrogen vapour-phase at the end of P1, resuscitated and experimental work performed at P4. Purity of MSC cultures was confirmed by immunophenotype and differentiation capacity; at the same passage as experimentation (P4) and according to international guidelines (45). Confirmatory immunophenotyping was achieved on all samples using the Human MSC Phenotyping Kit (Miltenyi Biotec, Woking, UK) according to manufacturer’s instructions, staining positively for CD73-APC, CD90-FITC and CD105-PE, and negatively for CD14-PerCP, CD20-PerCP, CD34-PerCP and CD45-PerCP (data not shown). MSC from three independent cultures (011B, 016, 025) were also induced to undergo tri-lineage differentiation using StemPro® Differentiation Kits (Thermo Fisher Scientific) for adipogenesis, chondrogenesis and osteogenesis, according to manufacturer’s instructions (data not shown).

#### Leukaemic-stromal co-cultures.

**Fig. 1.**
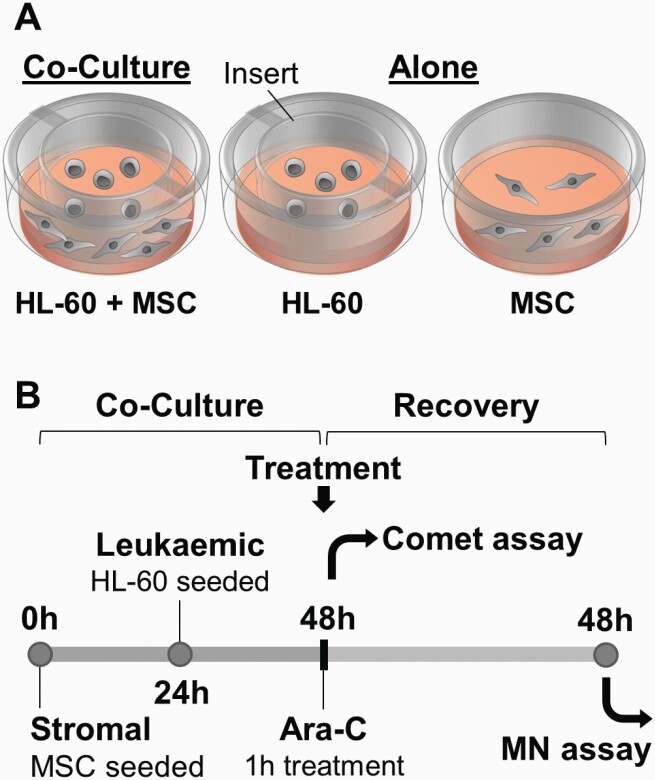
Schematic of co-culture and treatment for genotoxicity assessments. Cells were cultured alone or in co-culture, separated by a cell culture insert (A). Following 24 h in co-culture (or culture alone), both MSC and HL-60 cells were treated with ara-C and cells harvested for comet assays, or returned to culture in fresh medium for a recovery period, after which both MSC and HL-60 cells were harvested for micronucleus (MN) assays (B).

Patient MSC (5 × 10^4^) were seeded in 3.8-cm culture plates and allowed to adhere for 24 h. The AML cell line, HL-60 (ATCC® CCL240™) (1 × 10^5^), was seeded into 0.4 µm pore hanging cell culture trans-well inserts (Millipore UK Ltd., Watford, UK) above the stromal layer in the co-culture model (Figure 1). HL-60 were selected due to previously determined ara-C sensitivity (46) and to represent a constant HSC variable in the model. Cells were co-cultured for 24 h, with addition of 25 µM ara-C for the final 1 h, relevant to the clinical standard dose of 100–200 mg/m^2^ (47). Dose conversion was calculated based on an average person weighing 70 kg, equivalent to 50 l total body volume and 1.79 m^2^ total surface area. Cells were separated from co-culture and manually counted with addition of 0.4% trypan blue, from which cell viability, population doubling (PD) and relative increase in cell count (RICC) were determined, as per OECD guidelines prior to genotoxicity analysis (48) and detailed by Fellows *et al.* (49).

#### Micronucleus assay.

Cells were washed following co-culture and treatment and returned to culture in fresh medium for 48 h as per OECD guidelines for micronucleus (MN) assays without Cytochalasin B, requiring 1.5–2 cell divisions post-treatment. Cells were harvested, washed and 5 × 10^4^ cells in 150 µl phosphate buffered saline (PBS) loaded into Shandon™ Cytofunnel™ (Life Technologies, Inchinnan, UK) and mounted to polished glass slides by Cytospin™ 4 (Life Technologies) centrifugation at 1000 rpm for 10 min with high acceleration. Mounted cells were fixed using 100% methanol for 8 min with washes in phosphate buffer (pH 6.4–6.5; 0.66% KH_2_PO_4_, 0.33% Na_2_HPO_4_). For staining, slides were dipped briefly in phosphate buffer before exposure to 0.12 mg/ml Acridine Orange for 45 s, then finally washed in phosphate buffer. Slides were imaged by fluorescence microscopy (Nikon Eclipse 80i; Nikon, Tokyo, Japan) and NIS-Elements software (Nikon) under triple band-pass filter and manually scored for mononucleated, MN, binucleated, apoptotic, lobed, notched and multinucleated cells.

#### Alkaline comet assay.

Co-cultured cells (5 × 10^4^) were washed in PBS and 40-µl mounted onto Gelbond® film (Lonza, Slough, UK) in 0.5% low-melt agarose. Positive control gels were prepared simultaneously by treatment with 50 µM H_2_O_2_ for 10 min. All gels were lysed (2.5 M NaCl, 100 mM EDTA disodium salt, 10 mM Tris, 1% Triton X-100, 10% DMSO) for >1 h and exposed to alkaline electrophoresis buffer (pH 13; 1 mM EDTA, 300 mM NaOH) for 20 min to unwind the DNA. Gels were electrophoresed for 20 min (1 V/cm, 300 mA) and washed in neutralisation buffer (pH 7.5; 0.4 M Tris). Immediately prior to imaging, slides were stained with 20 µg/ml propidium iodide and cells imaged by fluorescence microscopy (Nikon 80i) under Texas red filter and NIS-Elements software (Nikon) and scored for % DNA in the tail (tail intensity %) using Comet Assay IV software (Perceptive Instruments Ltd., Bury St. Edmunds, UK). Further details of experiment protocols can be found in the supplement, as per the Minimum Information for Reporting on the Comet Assay (50).

### Statistical analysis

All statistical tests were performed in GraphPad Prism 9. One-way ANOVA was performed for comparison of three or more groups, followed by a *post hoc* test to define differences between groups. *T*-test was used to define differences between two groups. In all cases, statistical significance was assumed where *P* < 0.05. For each patient co-culture, MN incidence was calculated as a % of total scored cells, from which the % change between alone and co-culture groups, or mean MN incidence, was plotted for analysis. The median comet tail intensity (%) was calculated for each patient co-culture, representing the experimental unit (51). The % change between alone and co-culture groups, or mean tail intensity, was then plotted from median values for analysis. In the MN assay, 2000 cells were scored for each treatment group, while 200 cells were scored for the comet assay, whenever possible, as recommended by the genotoxicology testing guidelines in place when experimental work was undertaken (48,50,52).

## Results

### Primary BM cells from chemotherapy-treated patients have reduced *ex vivo* survival and those which did not expand *ex vivo* have increased genotoxicity

**Fig. 2.**
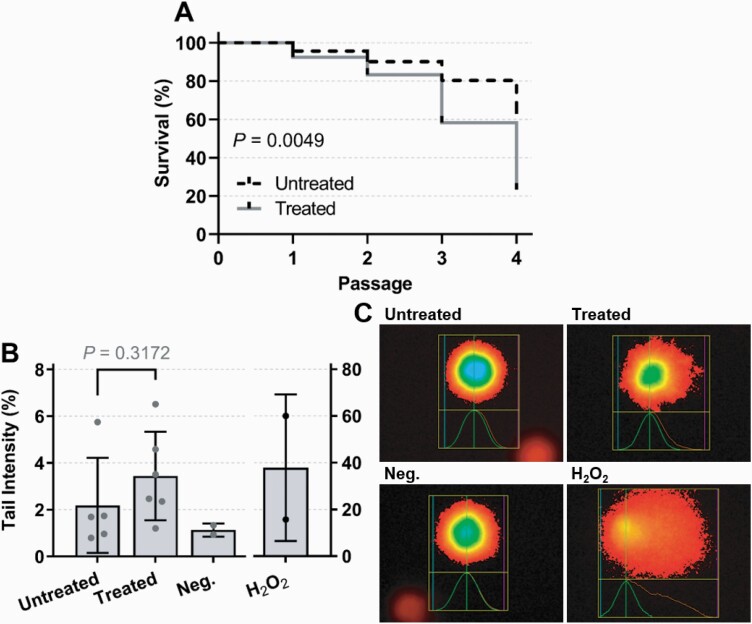
Survival of primary BM cultures and genotoxicity of those which did not expand *ex vivo*. Primary BM-MNC were cultured in standard conditions. (A) Kaplan–Meier survival was plotted for cultured BM cells from patients previously untreated/at diagnosis (*n* = 23; dashed line) or treated with chemotherapy for a haematological malignancy (*n* = 10; solid line) from passage 0 to 4. (B) Patient cultures which did not expand were categorised into untreated or previously treated, prior to assessment of comet tail intensity, alongside negative (Neg.; untreated HL-60 cells) and positive (HL-60 treated with H_2_O_2_) controls. Bars represent mean (± SD) of median tail intensity (%) from each experimental unit (patient culture). Untreated; *n* = 5 (mean 202 cells scored per patient culture), treated; *n* = 6 (mean 175 cells scored per patient culture). (C) Examples of scored cells for each experimental group (Comet IV software). Untreated; ID 027, Treated; ID 028.

The influence of genotoxicity from prior chemotherapy and *ex vivo* cell survival was assessed in primary patient BM cells. Patient BM-MNC cultures from previously treated individuals were significantly less likely to expand *ex vivo*, with survival at P4 (the point at which MSC cultures are considered ‘pure’ by standard isolation methods, and can therefore be utilised in experiments) significantly lower than samples taken from patients at diagnosis (*P* = 0.0049; Figure 2A). This was hypothesised as extensive impact on cellular function from the patient’s disease and/or prior treatment. The cultures which did not expand *ex vivo* were analysed by the alkaline comet assay, with more damaged DNA seen within the tail in cells from individuals who had previously been treated with chemotherapy, as compared to untreated patients at diagnosis (*P* = 0.3172; Figure 2B and C).

### Primary MSC may be sensitised to ara-C-induced genotoxicity in co-culture with leukaemic HL-60 cells, while the leukaemic cells appear protected

A co-culture system was designed to enable study of bidirectional effects, following *in vitro* ara-C treatment of primary MSC and a leukaemic cell line (representing a constant HSC variable; Figure 1A). This co-culture model was utilised to mimic the haematopoietic tumour microenvironment, whereby malignant HSC (HL-60) and MSC interact in the BM via soluble factors, as one such crosstalk mechanism (2,53).

Genotoxicity was investigated by the measurement of division abnormalities in the MN assay, following a 48-h recovery period post-treatment *in vitro* (Figure 1B), in accordance with the OECD guidelines which recommend testing after 1.5–2 PD (48) (Table 2). Ara-C treatment equivalent to standard *in vivo* dose (100–200 mg/m^2^) was utilised for a 1-h period to ensure that cytotoxicity did not exceed OECD recommended levels, with maximum cytotoxicity/viability reduction of 28.9% following recovery (Table 2). In MN assessments omitting the use of Cytochalasin B, as in this study, it is recommended to use RICC as a measure of cytotoxicity (54), with relevant data for cell cultures used in this study shown in Table 2. Mean RICC was acceptable (>55% ± 5%) for co-cultured HL-60 cells, MSC cultured alone and in co-culture; however, HL-60 cells cultured alone had mean RICC < 55% ± 5% (42.4%; Table 2).

**Table 2.** Mean genotoxicity and corresponding cytotoxicity of ara-C treatment in patient MSC and HL-60 co-cultures

Group	Ara-C (µM)	Mean MN % (SD)	Viability 1 h + 1.5–2 PD% (SD)	RICC (SD)	PD (SD)	Mean scored (MN)	Mean comet tail intensity % (SD)	Viability 1 h + 0 h % (SD)	Mean scored (comet)	*N*
HL-60 alone	0	2.47 (0.79)	92.8 (4.0)	100 (0.0)	3.7 (2.4)	1923	3.26 (2.26)	87.1 (7.0)	214	22
	25	5.76 (1.25)	71.1 (11.5)	42.4 (25.9)	1.7 (0.5)	1689	4.07 (3.38)	85.7 (8.6)	201	22
HL-60 co-culture	0	—	92.5 (3.3)	100 (0.0)	3.5 (1.6)	—	2.67 (1.94)	86.3 (7.0)	208	22
	25	4.91 (1.77)	84.2 (11.1)	60.0 (37.0)	2.4 (2.1)	1608	3.68 (2.89)	86.2 (8.4)	208	22
MSC alone	0	2.52 (1.19)	80.5 (9.7)	100 (0.0)	1.8 (0.6)	1534	3.44 (1.91)	82.0 (10.2)	190	22
	25	3.58 (1.69)	82.4 (10.3)	96.9 (63.3)	1.7 (0.7)	1571	4.95 (2.57)	81.7 (8.2)	169	22
MSC co-culture	0	—	78.4 (10.9)	100 (0.0)	1.7 (0.8)	—	3.65 (2.16)	80.1 (8.4)	182	22
	25	3.83 (1.79)	76.7 (11.6)	95.0 (74.0)	1.7 (0.7)	1346	5.86 (2.84)	80.3 (10.6)	193	22

MN, micronucleus; PD, population doubling; RICC, relative increase in cell count.

**Fig. 3.**
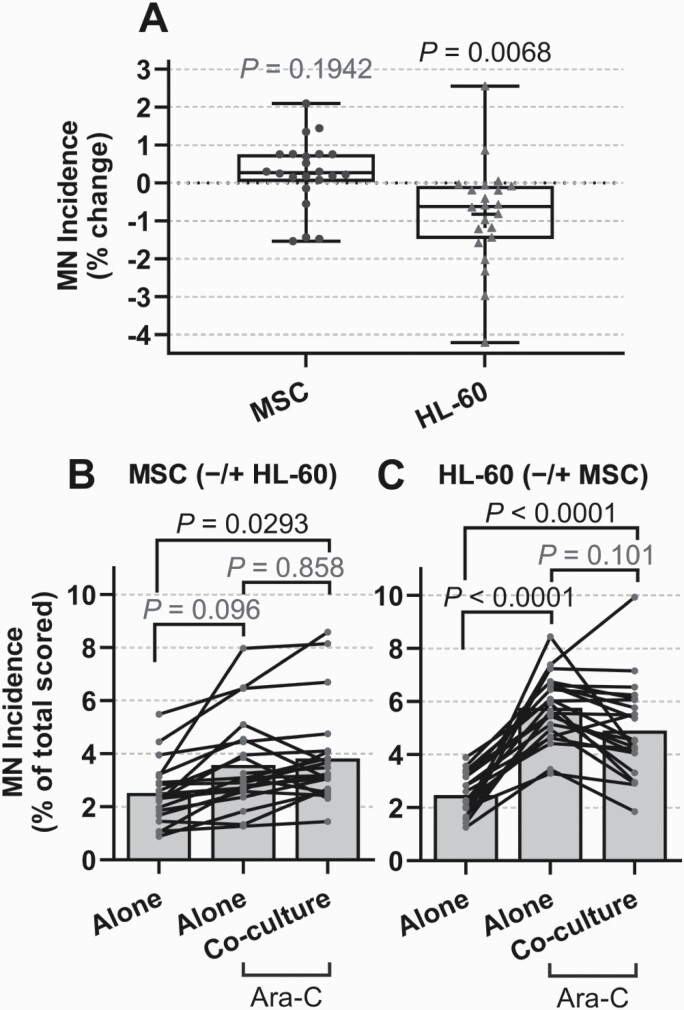
Genotoxicity in MSC/HL-60 in co-culture following *in vitro* ara-C treatment. Primary MSC and HL-60 cells were cultured alone or in co-culture ±) using inserts for 24 h prior to treatment with ara-C (25 µM) for 1 h. The MN assay was performed on cells after a further 1.5–2 PD (48 h). Data represent MN incidence; as % change of ara-C-treated cells between alone and co-culture, box and whisker with min and max of all data points (A); as % of total scored cells, for untreated cells alone, treated cells alone, and treated cells following co-culture (B/C), for MSC (± HL-60) (B) and HL-60 (± MSC) (C). Bars represent the mean MN incidence from each experimental unit (patient culture) (B and C). Data shown for independent co-cultures of HL-60 and MSC samples from patients (*n* = 22), where for each patient sample co-culture experiment, 2000 cells were scored per treatment group where possible (mean number of cells scored shown in Table 2).

The effect of MSC-HL-60 co-culture and ara-C treatment on genotoxicity was assessed in leukaemic-stromal co-cultures by measurement of MN incidence (Figure 3). In MSC, the MN incidence in *in vitro* ara-C-treated cultures was shown to be marginally altered overall, with a mean % change of 0.255% from MSC cultured alone to co-culture with HL-60 (*P* = 0.1942; Figure 3A). However, MN incidence in these ara-C-treated HL-60 cells was significantly reduced following co-culture with patient MSC, with a mean % change of −0.827% from culture alone to co-culture (*P* = 0.0068; Figure 3A). This indicates for the first time that leukaemic HSC may be protected by MSC from ara-C-induced genotoxicity.

The heterogeneity of MN as a measure of genotoxicity in these co-cultures was assessed in MSC (Figure 3B) and HL-60 cells (Figure 3C). Treatment with a physiological dose of ara-C only minimally increased MN incidence in MSC from all individuals analysed (Figure 3B, *P* = 0.096). Following *in vitro* ara-C treatment, MN incidence in MSC was further increased between untreated MSC cultured alone and when co-cultured with HL-60 cells; an effect seen in 17 of the 22 co-cultures (*P* = 0.0293; Figure 3B). This increase in MN between treated MSC cultured alone and in co-culture did not reach statistical significance overall (*P* = 0.858; Figure 3B). This demonstrates heterogeneity of MSC response to ara-C and malignant HSC influences, as well as some resistance against the genotoxic effects of ara-C subjection. Conversely, *in vitro* ara-C treatment significantly increased MN incidence in all HL-60 cultures, both in mono-culture (*P* < 0.0001) and co-culture with MSC (*P* < 0.0001), with mean 2.33-fold and 1.99-fold increase, respectively (Figure 3C). This increase was, however, attenuated by co-culture with the vast majority of primary MSC samples with a 1.17-fold reduction (*P* = 0.101). This resulted in a statistically significant reduction in MN % change between treated HL-60 alone and in co-culture with MSC (*P* = 0.0068; Figure 3A). These results demonstrate both ara-C sensitivity in HL-60 cells and some preservation by MSC.

**Fig. 4.**
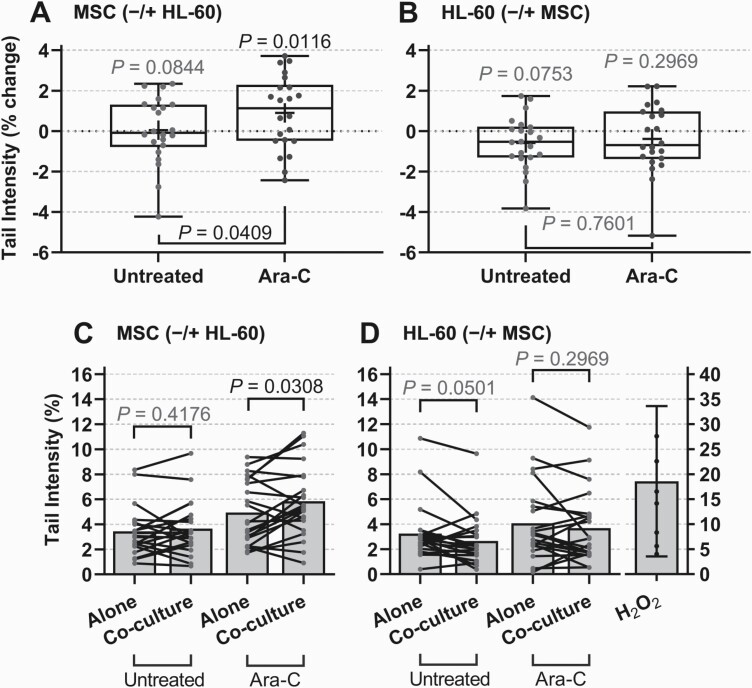
Genotoxicity in MSC/HL-60 following co-culture and *in vitro* ara-C treatment. Primary MSC and HL-60 cells were cultured alone or in co-culture using inserts for 24 h prior to treatment with ara-C (25 µM) for 1 h. Genotoxicity was determined by alkaline comet assay. Data represent comet tail intensity; as % change of untreated or ara-C-treated cells between alone and co-culture, box and whisker with min and max of all data points, for MSC (± HL-60) (A) or HL-60 cells (± MSC) (B); or as median % tail intensity for untreated or ara-C-treated cells alone, or following co-culture (C and D) for MSC (± HL-60) (C) and HL-60 cells (± MSC) (D). Bars represent mean of median tail intensity (%) from each experimental unit (patient culture). Data shown for independent co-cultures of HL-60 and MSC samples from patients (*n* = 22), where for each patient sample co-culture experiment, 200 cells were scored per treatment group where possible (mean number of cells scored shown in Table 2).

Genotoxicity was also assessed by the alkaline comet assay in MSC and HL-60 cells following treatment and co-culture (Figure 4), as MN formation and DNA fragmentation are recognised gold-standard genotoxicity measures in mammalian cells (55). In order to account for high variation between individuals, median % change in tail intensity between alone and co-culture was calculated. Comet tail intensity in untreated MSC was shown to not change following co-culture with HL-60, with a mean change of 0.0454% (*P* = 0.0844; Figure 4A). However, MSC treated with ara-C in co-culture with HL-60 showed a significant increase in tail intensity, with a mean change of 0.902% in co-culture (*P* = 0.0116; Figure 4A). Interestingly, MSC were sensitised to ara-C-induced genotoxicity with HL-60 as compared to untreated co-culture (*P* = 0.0409). Both untreated and ara-C-treated HL-60 showed a mean reduction in tail intensity when co-cultured with MSC (−0.5858%, *P* = 0.0753 and −0.3897%, *P* = 0.2969, respectively; Figure 4B), with no significant difference between the effect of co-culture in *in vitro* untreated and ara-C-treated cells (*P* = 0.7601).

Visualisation of changes in genotoxic response following co-culture in individual patient co-cultures demonstrated evident heterogeneity in both MSC (Figure 4C) and HL-60 (Figure 4D) response. Tail intensity was shown to increase in only 10 of 22 untreated MSC co-cultures (*P* = 0.4176) and 14 of 22 ara-C-treated co-cultures with HL-60 (*P* = 0.0308; Figure 4C), demonstrating a significant increase in tail intensity when MSC were co-cultured with HL-60 and ara-C-treated. In HL-60 cells, tail intensity was decreased in 14 of 22 untreated co-cultures (*P* = 0.0501) and 12 of 22 ara-C-treated co-cultures (*P* = 0.2969; Figure 4D). Variability in genotoxicity modulation highlights the challenge of distinguishing the impact of disease itself on the BM microenvironment

Sub-analyses of genotoxicity measures following co-culture of patient MSC and HL-60 cells were performed for the effect of prior chemotherapy treatment and the effect of patient AML/MM diagnosis (Supplementary Figure 1, available at *Mutagenesis* Online). Prior chemotherapy treatment was classified as those untreated due to no HM (no HM), those untreated at diagnosis for a HM (HM untreated) and those who had received prior chemotherapy treatment for a HM (HM treated) (Supplementary Figure 1A, available at *Mutagenesis* Online). Overall, the most marked effects between cells cultured alone to co-culture were seen in MSC from HM untreated patients co-cultured with HL-60 cells, and in HL-60 cells co-cultured with MSC from no HM patients (Supplementary Figure 1A, available at *Mutagenesis* Online). AML and MM patient cells were the focus of patient diagnosis assessment due to particularly poor *in vitro* cell survival (Figure 2; Supplementary Table I, available at *Mutagenesis* Online). Overall, an increase in ara-C genotoxicity in MSC by co-culture with HL-60 was similar when from AML and MM patients; however, HL-60 cells were greater protected when co-cultured with AML–MSC than with MM–MSC (Supplementary Figure 1B, available at *Mutagenesis* Online).

Together, these data suggest for the first time that HM patient MSC may be sensitised to ara-C genotoxicity by malignant HSC, while malignant HSC appear protected from the same effects, however, with heterogeneous responses mimicking those known clinically.

## Discussion

This study aimed to evaluate the effect of chemotherapy exposure and co-culture of primary BM–MSC and leukaemic cells on genotoxicity, in both cell types. Modulation of chemotherapy agents’ genotoxic effects in MSC is a meaningful clinical issue. Secondary malignancy complications are prominent post-treatment for a HM (26,56,57), and poor haematopoietic function can have catastrophic impact on the ability to continue treatment and can, therefore, reduce the patient’s quality of life and survival outcome (58).

Some communications in the field suggest MSC to be a robust and drug-resistant cell type (36,59,60), which must be partly the case as MSC withstanding myeloablative chemotherapy are not replaced in the BM niche by donor MSC post-alloSCT (35,61). On the contrary, MSC are known to be damaged by a host of chemotherapeutic agents (44,62), importantly with impairments to normal function in the BM (33,34,63). Altered expression of adhesive CD44 (63) and morphological changes to MSC (33) have been shown post-exposure to chemotherapy, including cyclophosphamide and melphalan, commonly used in treatment of HMs. Our group have previously shown persisting genotoxicity in both the HS-5 stromal cell line and cord blood MNC (which are partially MSC constituted) following cyclophosphamide treatment in appropriate metabolic models (34). In another study, BM-MSC and colony-forming unit fibroblast numbers were reduced up to one year post-SCT, where patients received high-dose ara-C consolidation chemotherapy and myeloablative busulfan/cyclophosphamide to prepare for the incoming graft (64). Our group further demonstrated persistence of genotoxic damage in patient MSC, even up to 17 years following cessation of chemotherapy for a HM (34).

These findings compliment those of this pilot study, whereby primary MSC which did not expand *in vitro* had significantly lower survival and increased genotoxicity when from patients who previously received *in vivo* chemotherapeutic treatment (Figure 2); potentially explained by defects in DNA damage response (DDR), replication stress pathways or genomic instability/DNA damage (65). Poor survival of cells from heavily treated individuals was overcome experimentally to some extent by performing the comet assay on failing cultures in the first passage (Figure 2). These failed cultures (*n* = 11), subsequently analysed for genotoxicity, originated from both treated (*n* = 5) and untreated patients (*n* = 6), demonstrating that factors beyond just chemotherapeutic damage are at play in MSC health. Notably, cells from patients with AML and MM made up ten of the 11 failed cultures (Supplementary Table I, available at *Mutagenesis* Online), both diseases with significant BM involvement. Damaged MSC may harbour mutations and have impaired ability to support normal haematopoiesis (33), inducing these secondary malignancies and BM failure in surviving patients (25). Reduced microenvironment function and consequent haematopoietic failure may ultimately explain poor therapeutic efficacy in patients with haematological disease and warrants further research.

The effect of co-culture of MSC and malignant HSC on ara-C-induced genotoxicity was assessed by measurement of MN formation (Figure 3) and DNA fragmentation (Figure 4), using a trans-well model to replicate bidirectional leukaemic-stromal interactions (Figure 1). Primary patient MSC (the variable of focus) were co-cultured with the HL-60 cell line, since leukaemic cell lines do not require the support of a stromal feeder layer for survival *in vitro*, unlike primary HSC/leukaemic cells (66,67). Cells in the co-culture model were treated with the nucleoside analogue clastogen ara-C (24), commonly administered in combined therapies for acute leukaemias and lymphoma (68). Ara-C inhibits the gap-filling step of excision repair, resulting in single-strand breaks at these sites and chromosome breaks/MN at the next cellular division (69,70). DNA repair and the rejoining of DNA strand breaks formed by other agents are also inhibited (71), as well as inhibiting polymerases during normal DNA replication (71). This agent, therefore, was used to assess the modulation of genotoxicity by MSC and malignant HSC in co-culture, by measure of MN formation and DNA fragmentation.

A significant decrease in MN incidence in HL-60 cells following co-culture with MSC (Figure 3A; *P* = 0.0068) indicates for the first time that leukaemic cells may be protected from ara-C-induced genotoxicity by MSC. Our pilot study also showed a trend of decreased tail intensity overall in HL-60 following co-culture with MSC (Figure 4B and D). This concurs with one previous study showing BM-MSC to provide protection of the U266 MM cell line from melphalan-induced genotoxicity (72). There are few studies of this kind on genotoxicity modulation, while numerous reports have shown that the BM is able to provide protection from the cytotoxic effects of a number of agents at a cellular level (13,38–42,53). There was also a non-significant increase in MSC MN incidence following co-culture with HL-60 (Figure 3), while comet tail intensity significantly increased in MSC following *in vitro* ara-C treatment and co-culture with HL-60 (Figure 4A; *P* = 0.0116, Figure 4C; *P* = 0.0308). Another study showed that the ability of MSC to protect leukaemic cells was reduced following ara-C treatment with a cytotoxicity perspective (40), however, research in the area of genotoxicity are lacking, requiring further research to elucidate potential mechanisms altering genotoxicity response by leukaemic and stromal cells. One study showed ATM-dependent DDR to induce IL-6 secretion by BM stromal cells and subsequent myeloma cell chemoresistance (73); probing for the specific DDR cascade may reveal targetable mechanisms for future HM therapies (65).

Treatment of cells for co-culture experiments for 1 h at 25 µM was selected in line with therapeutic standard dose (100–200 mg/m^2^) for a duration that was within cytotoxic limits for genotoxicity testing (48,52). Best efforts were made to ensure cytotoxicity was at an acceptable level for minimisation of potential false-negative and -positive genotoxicity results; viability and RICC > 55 ± 5% (48). Cytotoxicity was acceptable in all experimental groups, while RICC was acceptable in all groups bar HL-60 cells cultured alone, which may increase the possibility of false-negative genotoxicity results in this group (Table 2). Harvesting cells for the MN assay at 48 h post-treatment was used for consistency across experimental groups, with PD between 1.7 and 3.7 (guidelines recommend 1.5-2 PD). Additionally, as prior chemotherapy treatment in patients significantly hindered BM cell survival *in vitro*, and that these BM samples had to be cultured to P4 for MSC purity, there were limited cell numbers for multiple co-culture assessments. Therefore, some of the 22 patient co-cultures had lower than optimal cells for MN and comet analyses (Table 2). Genotoxicity data in this study should, therefore, be interpreted with these limitations in mind, with further studies investigating extended ara-C treatment times at lower doses and post-treatment recovery prior to comet analysis, where cell numbers allow. Whilst small changes were seen in terms of increased genotoxicity in MSC and reduced genotoxicity in HL-60, we do not know the true physiological impact. Treatment in *in vitro* co-culture models is likely to underestimate effects seen *in vivo* with chemotherapeutic agents, especially as regimens for HMs, particularly AML, feature a combination of agents with different mechanisms of action. Additionally, data from co-culture studies are likely to be skewed towards the ‘least damaged’ MSC samples, in cells capable of survival to P4.

Sub-analysis of genotoxicity in co-cultures showed minimal alteration in MSC by prior *in vivo* chemotherapeutic treatment and AML/MM diagnosis (Supplementary Figure 1, available at *Mutagenesis* Online). However, in HL-60 cells, the most marked protection was in co-culture with MSC from untreated patients without HM as compared to HM untreated/treated patient MSC, and with AML–MSC as compared to MM–MSC (Supplementary Figure 1, available at *Mutagenesis* Online). The degree of MSC resistance to damage is debated in the literature, with some studies showing functional damage to MSC (33,34) and differential mechanisms in support of malignancy (13,43,44), while others show resistance to chemotherapy (36,59,60). This further adds to the emerging picture that damaged MSC communicate in an aberrant manner; however, variations in findings in the literature also highlight the complexity of such mechanisms in the BM.

This pilot investigation suggests ‘healthy’ untreated BM-MSC to maximally protect malignant HSC from *in vitro* genotoxicity, perhaps suggesting aberrant MSC functionality by presence of a HM and by previous chemotherapy treatment in patients. One previous study showed damaged MSC to be less equipped to support HSC growth and migration *in vitro* (33). Additionally, work by Somaiah *et al.* also showed pre-treated MSC to prevent the efficacy of ara-C, daunorubicin and vincristine against HL-60 as compared to untreated MSC (44).

Investigations into MSC in the HM setting are complex as these diseases, AML and MM in particular, are known to invoke significant changes in the BM microenvironment (74,75). Distinguishing the impact of disease on the BM microenvironment and malignant cells is also challenged by biological variation, demonstrated by high heterogeneous response in the co-culture model reflecting issues of both inter- and intra-patient heterogeneity in disease diagnosis and treatment seen clinically (9,10,76,77). Variations in cell behaviour experimentally from AML patients has additionally been documented in a number of other studies (14,41,77–79), with compounding factors of our pilot cohort including a mix of malignancies, variations in donor age, gender, treatment received (Supplementary Table I, available at *Mutagenesis* Online); consequently life-time genotoxic exposures. Future experimentation using this model may also benefit from comparing the cellular response between co-culture and cells alone, where alone groups use the same cell type cultured in both compartments. Investigating direct co-culture effects as well as probing for the specific signals conveyed in our indirect model would also be an interesting next step. One previous study showed that soluble factors (not microvesicles or exosomes) secreted by stromal cells were responsible for leukaemic cells protection from ara-C-induced cytotoxicity (53).

These pilot investigations deserve further work in larger patient cohorts to confirm the effects seen, as samples were indeed limited in the small centre where BM samples were sources, particularly of some rarer HM subtypes (Supplementary Table I, available at *Mutagenesis* Online). The feasibility of such a study with finite patient material was confirmed as a secondary aim. Studies in this area are further limited by the availability of healthy controls, since the ‘no HM’ group in this study cannot be truly considered as such due to the nature of the individual undergoing BM investigations for a potential disorder. Malignancies such as AML, while crudely grouped by subtype for treatment purposes, have been acknowledged as unique to each individual due to complex genetic aberrations (21,80). The very fact that biological and experimental heterogeneity are hallmarked in HM investigations, adds to the relevance of the results of this study, which showed some statistically significant genotoxicity modulation despite such challenges.

Overall, this pilot study showed reduced *ex vivo* survival and increased genotoxicity in primary MSC from patients who had undergone chemotherapy treatment for a HM, as compared to patient cells at diagnosis. Critically, MSC showed some protection of leukaemic HL-60 from spontaneous and ara-C-induced genotoxicity, with concurrent increases in MSC genotoxic damage in the presence of leukaemia cells. These investigations of altered genotoxicity in the leukaemic microenvironment are not documented elsewhere in the literature to our knowledge, despite the evident impact of lasting damage in surviving haematology patients. Additionally, the variation in patient results reflects both clinical and biological heterogeneity, which challenge the management of HMs. This study provides the basis for future research to investigate the mechanisms of such bidirectional effects, including the impact of treatment and disease. Future work may impact long-term therapeutic efficacy by revealing how to reduce genotoxicity and treatment resistance—some of the foremost issues in HMs and AML specifically today.

## Supplementary Material

geab033_suppl_Supplementary_Figure_S1

geab033_suppl_Supplementary_Table_S1

geab033_suppl_Supplementary_Material
