# Supplementary material for: Primary mesenchymal stromal cells in co-culture with leukaemic HL-60 cells are sensitised to cytarabine-induced genotoxicity, while leukaemic cells are protected
Source: Mutagenesis. 2021 Sep 10;36(6):419–28. doi: 10.1093/mutage/geab033 (PMC8633936; doi:10.1093/mutage/geab033)
Supplement: geab033_suppl_Supplementary_Figure_S1 [file geab033_suppl_supplementary_figure_s1.docx]

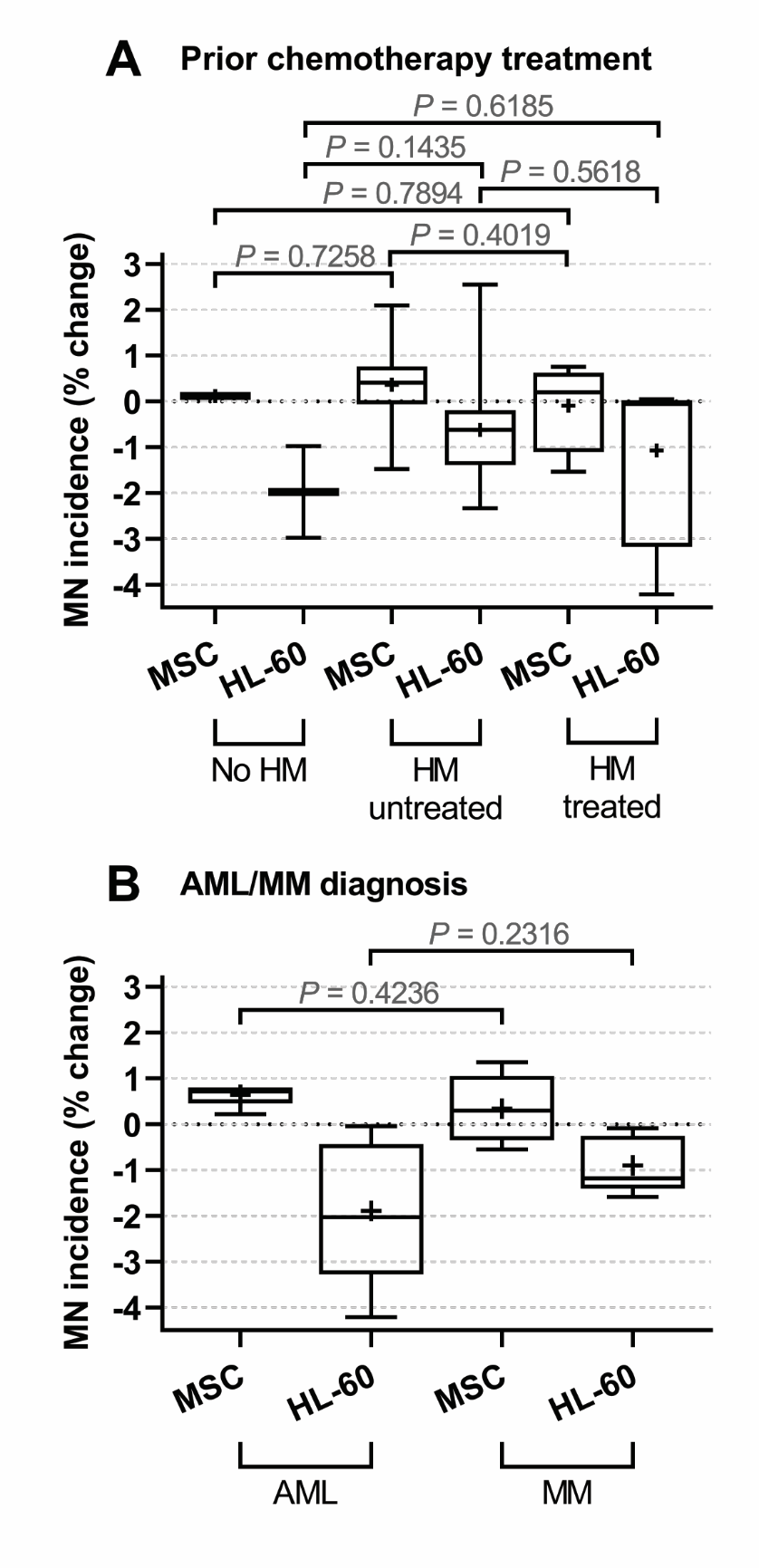


**Supplementary Fig. 1.** Genotoxicity of MSC and HL-60 following co-culture and *in vitro* ara-C treatment, grouped by previous treatment or diagnosis group. Primary MSC and HL-60 cells were cultured alone or in co-culture for 24 h prior to treatment with ara-C (25 µM) for 1 h. The MN assay was performed on cells after a further 1.5-2 PD (48 h). Data represent MN incidence; as % change of ara-C-treated cells from alone and co-culture; box and whisker with min and max of all data points. Effects of patient prior treatment was assessed (A), representing independent co-cultures of HL-60 with MSC from previously untreated patients found not to have a HM (no HM; *n* = 2), untreated patients at diagnosis (HM untreated; *n* = 16) and HM patients post-treatment (HM treated; *n* = 4). The effects of patient diagnosis were also assessed by diagnosis group (B), representing independent co-culture of HL-60 with MSC from patients with AML (*n* = 5) and MM (*n* = 5). For each patient sample co-culture experiment, 2,000 cells were scored per group where possible (mean number of cells scored shown in Table II of the main manuscript).
