# Supplementary material for: Primary mesenchymal stromal cells in co-culture with leukaemic HL-60 cells are sensitised to cytarabine-induced genotoxicity, while leukaemic cells are protected
Source: Mutagenesis. 2021 Sep 10;36(6):419–28. doi: 10.1093/mutage/geab033 (PMC8633936; doi:10.1093/mutage/geab033)
Supplement: geab033_suppl_Supplementary_Table_S1 [file geab033_suppl_supplementary_table_s1.docx]

**Supplementary Table I.** Patient characteristics for provided BM samples.

| ID No. | M/F | Age | Diagnosis | TX (Y/N) | Regimen | No Exp.  (X) |
| --- | --- | --- | --- | --- | --- | --- |
| 001 | F | 73 | Essential thrombocythaemia | N | – |  |
| 002 | F | 67 | Plasma cell myeloma | N | – |  |
| 003 | M | 74 | MDS-ring sideroblasts, single lineage dysplasia | N | – |  |
| 004 | F | 68 | Ph+ CML | N | – |  |
| 005 | F | 78 | Infective endocarditis (non-malignant) | N | – |  |
| 006 | F | 80 | Asymptomatic myeloma | N | – |  |
| 007 | M | 79 | Myelofibrosis | N | – |  |
| 010 | M | 69 | AML | N | – |  |
| 011* | M | 36 | AML, myelodysplasia related changes | N | – |  |
| 012 | M | 59 | Plasma cell myeloma | N | – |  |
| 014 | F | 73 | Plasma cell myeloma | N | – | X |
| 015 | F | 74 | Plasma cell myeloma | N | – | X |
| 016 | F | 52 | Plasma cell myeloma | N | – |  |
| 018 | F | 70 | Plasma cell myeloma | N | – |  |
| 019 | F | 73 | Leukaemic mantle cell lymphoma | N | – |  |
| 020 | M | 56 | Essential thrombocythaemia | N | – |  |
| 021 | M | 64 | Primary myelofibrosis accelerated phase (JAK2+) | N | – |  |
| 022 | F | 66 | JAK2+ myelofibrosis | N | – | X |
| 023 | M | 90 | Refractory anaemia, ring sideroblasts, unilineage dysplasia | N | – |  |
| 025 | M | 71 | AML | N | – |  |
| 027 | M | 68 | AML – FLT3 & NPM1 wild type | N | – | X |
| 030 | M | 77 | MDS transforming to AML (complex karyotype including del 5q, del17) | N | – | X |
| 031 | F | 57 | Immune thrombocytopenic purpura | N | – |  |

**Supplementary Table I.** Continued.

| ID No. | M/F | Age | Diagnosis | TX (Y/N) | Regimen | No Exp.  (X) |
| --- | --- | --- | --- | --- | --- | --- |
| 008 | F | 68 | APML in remission, MRD neg, breast cancer history | Y | C1- AIDA, Con1- AIDA, Con2- ATRA + Mitox, Con4- IDA. Anastrozole | X |
| 009 | F | 62 | Small lymphocytic lymphoma | Y | Radiotherapy left neck (4 Gy; 41 months prior), right parotid (4 Gy; 40 and 12 months prior) |  |
| 011B***** | M | 36 | AML, myelodysplasia related changes | Y | Cycle 1 AML19 Trial |  |
| 011C***** | M | 36 | AML, myelodysplasia related changes | Y | Cycle 2 AML19 Trial |  |
| 013 | M | 77 | IgG ĸ myeloma relapsing | Y | VCD (35 months previously) | X |
| 017 | M | 56 | AML 2° to ALL | Y | ALL- Ind1-2- DNR + vincristine + Dex + PEG-ASP + methotrexate, Intens, Con1-4, Maint, 3-monthly vincristine, methotrexate (11 months previously) (UKALL14 trial)  AML- C1 FLAG-IDA | X |
| 024 | M | 73 | Follicular lymphoma (stage IV), MDS/MPN/ chronic anaemic BM | Y | C1-6- R-CVP (15 months previously), Maint- rituximab |  |
| 026 | M | 39 | AML (FLT3-ITD mut.) | Y | C1-2- ara-C + DNR + midastaurin | X |
| 028 | M | 67 | APML | Y | ATRA + ATO (molecular complete remission) | X |
| 029 | M | 67 | Myeloma (IgG Kappa) | Y | CCD, cyclophosphamide primed stem cell collection, HD melphalan and stem cell return, Maint- carfilzomib (CARDAMON trail) | X |

* matched samples from patient pre- and post-treatment. M/F; Male/Female. No Exp.; No Expansion. TX; Treated. Samples marked with ‘X’ did not expand *in vitro* and were used for initial genotoxicity assessments, with remaining samples assessed in co-culture experiments.
