# Supplementary material for: Primary mesenchymal stromal cells in co-culture with leukaemic HL-60 cells are sensitised to cytarabine-induced genotoxicity, while leukaemic cells are protected
Source: Mutagenesis. 2021 Sep 10;36(6):419–28. doi: 10.1093/mutage/geab033 (PMC8633936; doi:10.1093/mutage/geab033)
Supplement: geab033_suppl_Supplementary_Material [file geab033_suppl_supplementary_material.docx]

Supplementary Methods

Cell culture

Sample collection

BM aspirate samples were obtained from patients undergoing routine investigations for the diagnosis or monitoring of a haematological disease at the Royal United Hospitals NHS Foundation Trust, Bath, following donor consent and NHS ethics approval (18/NI/0036). BM aspiration was performed by clinical professionals from the iliac crest and collected into sterile lithium heparin sample tubes. Samples were transported to UWE Bristol for processing, which was performed on the same day as receipt and as soon as possible following collection (2-72 h). Samples were maintained at room temperature from collection to processing for the greatest cell viability and yield.

Mononuclear cell isolation

The mononuclear cell (MNC) fraction was isolated from BM aspirates by density gradient centrifugation, as previously described (34). BM aspirate samples (1-5.5 mL) were diluted 1:1 with low glucose (LG; 1,000 mg/L) Dulbecco’s Modified Eagle’s Medium (DMEM) before gentle layering onto an equal volume of Histopaque-1077 (density 1.077 g/mL), performed at room temperature in 15 mL falcon tubes. Cells were centrifuged at 600 *xg* for 20 min without brakes. MNC fraction was collected into a separate tube and washed in MSC medium (LG-DMEM supplemented with 10% foetal bovine serum (FBS; STEMCELL Technologies, Cambridge, UK) and 2 mM L-glutamine). Residual erythrocytes were removed by resuspension in red cell lysis buffer (150 mM NH_4_Cl, 100 mM NaHCO_3_, 1.3 mM EDTA) for 5 min and washed in MSC medium. Cells were manually counted with addition of 0.4% trypan blue to determine viability.

MSC culture

MNC were seeded into 25 cm^2^ vented culture flasks at 4 x10^5^/cm^2^ in MSC medium and incubated at 37°C/5% CO_2_. After three days, non-adherent haematopoietic cells were removed and replaced with fresh medium, with cultures maintained by weekly demi-depletion until 70% confluency was reached. Cell monolayers were washed with PBS, incubated with trypsin (0.05% trypsin/0.02% EDTA) for up to 15 min to detach, centrifuged at 300 *x*g for 5 mins and reseeded in 25 cm^2^ culture flasks in MSC medium at 4 x10^3^/cm^2^ (passage one; P1) and for all further passages.

At the end of P1, cells were harvested for cryopreservation by resuspension in MSC medium containing 25% FBS and 10% DMSO and immediate storage at −80°C in a freezing container to ensure a cooling rate of 1°C/min. After 24 h, cells were transferred to liquid nitrogen vapour-phase storage. Cells were resuscitated in MSC medium and continued in culture as previously described, until the end of P4 when experimental work was performed. All cultures were treated in the same manner to reduce variability and account for differences in growth rate.

Purity of MSC cultures was confirmed by immunophenotype and differentiation capacity; at the same passage as experimentation (P4) and according to international guidelines (45). Confirmatory immunophenotyping was achieved on all samples using the Human MSC Phenotyping Kit (Miltenyi Biotec, Woking, UK) according to manufacturer’s instructions, staining positively for CD73-APC, CD90-FITC and CD105-PE, and negatively for CD14-PerCP, CD20-PerCP, CD34-PerCP and CD45-PerCP (data not shown). MSC from three independent cultures (011B, 016, 025) were also induced to undergo tri-lineage differentiation using StemPro® Differentiation Kits (Thermo Fisher Scientific) for adipogenesis, chondrogenesis and osteogenesis, according to manufacturer’s instructions (data not shown).

Leukaemic-stromal co-cultures

Patient MSC (5 x10^4^) were seeded in 3.8 cm culture plates and allowed to adhere for 24 h in MSC medium. The AML cell line, HL-60 (ATCC® CCL­240™) (1 x10^5^), was maintained as per supplier’s guidelines, in Roswell Park Memorial Institute 1640 medium supplemented with 10% FBS and 2 mM L-glutamine. HL-60 were seeded into 0.4 µm pore hanging cell culture trans-well inserts (Millipore UK Ltd., Watford, UK) above the stromal layer in the co-culture model (Figure 1) following 24 h. MSC and HL-60 cells in the co-culture model shared MSC medium. HL-60 were selected due to previously determined ara-C sensitivity (46) and to represent a constant HSC variable in the model. Cells were co-cultured for 24 h, with addition of 25 µM ara-C for the final 1 h, relevant to the clinical standard dose of 100-200 mg/m^2^ (Kadia *et al.*, 2015). Dose conversion was calculated based on an average person weighing 70 kg, equivalent to 50 L total body volume and 1.79 m^2^ total surface area. Cells were separated from co-culture, wells harvested by washing (HL-60) or trypsinisation (MSC) and manually counted with addition of 0.4% trypan blue, from which cell viability was calculated.

Micronucleus assay

Cells were washed following co-culture and treatment and returned to culture in fresh medium for 48 h as per OECD guidelines for micronucleus (MN) assays without Cytochalasin B, requiring 1.5-2 cell divisions post-treatment. Cells were harvested and manually counted with trypan blue, from which population doubling (PD) and relative increase in cell count (RICC) were determined, as per OECD guidelines prior to genotoxicity analysis (48) and detailed by Fellows *et al.* (49)*.* Cells were immediately washed in PBS and 5 x10^4^ cells in 150 µL phosphate buffered saline (PBS) loaded into Shandon™ Cytofunnel™ (Life Technologies, Inchinnan, UK) and mounted to ethanol polished glass slides by Cytospin™ 4 (Life Technologies) centrifugation at 1,000 rpm for 10 min with high acceleration. One slide was performed per experimental group. Mounted cells were fixed using 100% methanol for 8 min with washes in freshly prepared phosphate buffer (pH 6.4-6.5; 0.66% KH_2_PO_4_, 0.33% Na_2_HPO_4_). For staining, slides were dipped briefly in phosphate buffer before exposure to 0.12 mg/mL Acridine Orange (freshly prepared from stock solution) for 45 sec, then finally washed in fresh phosphate buffer. Slides were imaged by fluorescence microscopy (Nikon Eclipse 80i; Nikon, Tokyo, Japan) and NIS-Elements software (Nikon) under triple band-pass filter and manually scored for mononucleated, MN, binucleated, apoptotic, lobed, notched, and multinucleated cells. Slides were processed and stored protected from light to reduce damage and dye fading.

Alkaline comet assay

Cells were harvested and washed following co-culture and treatment in PBS at 4°C and immediately processed for comet assessment. A pellet of 1 x10^5^ cells was resuspended in 80 µL in 0.5% low-melt agarose (freshly prepared from powder) held at 37°C. Cells (40 µL containing 5 x10^4^) were mounted onto Gelbond® film (Lonza, Slough, UK), covered with a glass coverslip and set at 4°C protected from light. Coverslips were removed and gels submerged in chilled lysis buffer (2.5 M NaCl, 100 mM EDTA disodium salt, 10 mM Tris, 1% Triton X-100, 10% DMSO) for > 1 h at 4°C. Positive control gels were prepared simultaneously (untreated HL-60 cells) by treatment of cells mounted in agarose with 50 µM H_2_O_2_ for 10 min, washed and added to lysis buffer. All lysed gels were exposed to alkaline electrophoresis buffer (pH 13; 1 mM EDTA, 300 mM NaOH) in a pre-cooled (4°C) electrophoresis tank for 20 min to unwind the DNA. Gels were electrophoresed for 20 min (1 V/cm, 300 mA) and washed in neutralisation buffer (pH 7.5; 0.4 M Tris). Gels were stored in neutralisation buffer until imaging as soon as possible. Immediately prior to imaging, slides were stained by addition of 20 µL of 20 µg/mL propidium iodide, a glass coverslip and cells imaged by fluorescence microscopy (Nikon 80i) under Texas red filter and NIS-Elements software (Nikon), under X40 magnification. Images containing enough cells for scoring were saved for scoring, which was performed using Comet Assay IV software (Perceptive Instruments Ltd., Bury St. Edmunds, UK). Comet tail intensity was selected as the most appropriate measure.

Statistical analysis

All statistical tests were performed in GraphPad Prism 9 using non-parametric methods. One-way ANOVA was performed for comparison of three or more groups, followed by a *post-hoc* test to define differences between groups. T-test was used to define differences between two groups. In all cases, statistical significance was assumed where *P* < 0.05. For each patient co-culture, MN incidence was calculated as a % of total scored cells, from which the % change between alone and co-culture groups, or mean MN incidence, was plotted for analysis. The median comet tail intensity (%) was calculated for each patient co-culture, representing the experimental unit (50). The % change between alone and co-culture groups, or mean tail intensity, was then plotted from median values for analysis. In the MN assay, 2,000 cells were scored for each treatment group, while 200 cells were scored for the comet assay, whenever possible, as recommended by the genotoxicology testing guidelines in place when experimental work was undertaken (48,52).
